# The Prevalence of Insomnia Subtypes in Relation to Demographic Characteristics, Anxiety, Depression, Alcohol Consumption and Use of Hypnotics

**DOI:** 10.3389/fpsyg.2020.00527

**Published:** 2020-03-24

**Authors:** Ingrid Bjorøy, Vilde Aanesland Jørgensen, Ståle Pallesen, Bjørn Bjorvatn

**Affiliations:** ^1^Department of Global Public Health and Primary Care, University of Bergen, Bergen, Norway; ^2^Norwegian Competence Center for Sleep Disorders, Haukeland University Hospital, Bergen, Norway; ^3^Department of Psychosocial Science, University of Bergen, Bergen, Norway

**Keywords:** insomnia, insomnia subtypes, anxiety, depression, alcohol, hypnotics

## Abstract

**Objective:**

The aim of the present study was to examine the prevalence of insomnia subtypes in relation to several demographic characteristics, as well as to investigate the prevalence of possible anxiety and depression, alcohol consumption and use of hypnotics within the different insomnia subtypes.

**Methods:**

The present study was based on an extensive web-based survey made publicly available in 2012. The data was downloaded in January 2019, after 113 887 people had responded to parts of, or the entire questionnaire. The 64 503 participants who met the criteria for chronic insomnia disorder according to the Diagnostic and Statistical Manual of Mental Disorders, fifth edition (DSM-5) comprised the study population. The present study divided insomnia into seven subtypes based on type of sleep difficulty reported; sleep onset insomnia (SOL-insomnia), sleep maintenance insomnia (WASO-insomnia), early morning awakening insomnia (EMA-insomnia) and combinations of these. Data were analyzed with chi-square tests and logistic regression analyses adjusted for sex, age, level of education and marital status.

**Results:**

More than 60% of the study population met the criteria of either SOL-insomnia or a mixed insomnia subtype consisting of SOL-, WASO- and EMA-insomnia (SOL + WASO + EMA-insomnia). The percentage distribution of insomnia subtypes within the demographic characteristics showed that participants with female sex, high age, low level of education and who were divorced, separated or a widow/widower had a higher prevalence of SOL + WASO + EMA-insomnia compared to their respective demographic counterparts. The prevalence of possible anxiety, possible depression and use of hypnotics were higher among participants with SOL + WASO + EMA-insomnia compared to the other insomnia subtypes. The combination of WASO- and EMA-insomnia (WASO + EMA-insomnia) was associated with the most frequent alcohol consumption.

**Conclusion:**

Our findings suggest that there are major differences between the insomnia subtypes, both regarding demographics, but also in terms of how the complaints may affect daily life. Participants with combinations of SOL, WASO and EMA were more likely than participants with the other subtypes to have possible anxiety and possible depression, high alcohol consumption and to use hypnotics.

## Introduction

Insomnia is the most frequently occurring sleep disorder in the adult population (Morin et al., 2006), with a prevalence ranging from 5.8–20.0% when defined in accordance with formal diagnostic criteria (Ohayon, 2002; Pallesen et al., 2014; Riemann et al., 2017; Bjorvatn et al., 2018). Commonly identified risk factors for insomnia are female sex, high age, low level of education, and being separated, divorced or widowed (Ohayon, 2002; Taylor et al., 2005; Zhang and Wing, 2006; Atalay, 2011). Alcohol consumption and use of hypnotics are also related to insomnia (Ohayon, 2002; Atalay, 2011; Kim et al., 2017; Brooks et al., 2018). According to the criteria found in the fifth edition of the Diagnostic and Statistical Manual of Mental Disorders (DSM-5), insomnia disorder is defined as the subjective experience of difficulty initiating sleep, maintaining sleep and/or early morning awakening, occurring for at least three nights a week for a duration of ≥3 months. A further criterion is clinically significant distress or impairment in important areas (e.g., social, occupational, educational) of functioning (American Psychiatric Association [APA], 2013).

DSM-5 discarded the distinction between primary and secondary insomnia found in the previous version of the diagnostic system (American Psychiatric Association [APA], 2013) and rather focused on a more bidirectional relationship between insomnia and co-occurring medical and psychiatric disorders (Jansson-Frojmark and Lindblom, 2008; Sivertsen et al., 2012; Reynolds and O’Hara, 2013; Gupta et al., 2014). By contrast, the previous edition, DSM-IV, focused more on a causal relationship between coexisting disorders and insomnia. However, the latter conceptualization could lead to under-treatment of insomnia symptoms (National Institute of Health [NIH], 2005; Manber et al., 2008; Gupta et al., 2014). Previous studies have suggested that heterogeneity exists within patients with insomnia disorder (Blanken et al., 2019). Therefore, in addition to distinguishing between primary and secondary insomnia or insomnia with or without comorbidity, previous research has made repetitive attempts to further divide patients with insomnia into different subtypes, based on either sleep complaints or non-sleep characteristics (American Psychiatric Association [APA], 2000; American Academy of Sleep Medicine, 2005; Espie et al., 2012; Benjamins et al., 2017; Blanken et al., 2019). To our knowledge, no previous subtyping based on non-sleep characteristics has simplified the clinical management of insomnia sufficiently to warrant amendments in the clinical approach (Ferini-Strambi et al., 2019; Kitajima, 2019). Among others, this applies to a recent impressive study by Blanken et al. (2019) who divided insomnia into subtypes based on non-sleep characteristics such as life history, mood perceptions, and personality. Other insomnia subtypes reported in the literature have differentiated between insomnia with normal and short sleep duration (Miller et al., 2018), insomnia subtypes based on assumed etiology (e.g., psychophysiological, paradoxical, inadequate sleep hygiene) (American Academy of Sleep Medicine, 2005), data-driven/derived subtypes (Dekker et al., 2015) and subtypes based on consistency (persistent, remission, relapse) (Wu et al., 2014). The most commonly used subdivision seems to concern when in the main sleep period (sleep onset, maintenance, early morning awakening) the symptoms occur (Hohagen et al., 1994; Yokoyama et al., 2010). Overall, the most logical approach would be to subtype according to specific sleep-related characteristics. Therefore, in the present study we divided insomnia into subtypes based on when the sleep difficulty was experienced during the night (sleep onset insomnia, sleep maintenance insomnia and early morning awakening insomnia) which as noted, represent subtypes previously described in the literature (Taylor et al., 2005; Pigeon, 2010; Yokoyama et al., 2010; Espie et al., 2012; Ikeda et al., 2017). Even though there exist some research on demographic characteristics associated with insomnia, there is a dearth of research on the distribution of insomnia subtypes within these characteristics.

As mentioned, the relationships between insomnia and anxiety, and insomnia and depression are considered bidirectional (Jansson-Frojmark and Lindblom, 2008; Baglioni et al., 2010, 2011; Sivertsen et al., 2012; Alvaro et al., 2014; Dolsen et al., 2014). To be able to predict which insomnia patients who are more likely to suffer from psychiatric disorders, previous studies have examined the prevalence of depression in specific sleep-related subtypes (Rodin et al., 1988; Lustberg and Reynolds, 2000; Taylor et al., 2005; Yokoyama et al., 2010; Espie et al., 2012; Ikeda et al., 2017). However, there are inconsistencies on which subtype that most strongly has been associated with depression. Historically, early morning awakening was considered a characteristic of depression in the field of psychiatry (World Health Organization [WHO], 1992) and several studies support this notion (Rodin et al., 1988; Lustberg and Reynolds, 2000). However, both Yokoyama et al. (2010), and Ikeda et al. (2017) found that difficulty initiating sleep is more strongly associated with depression than early morning awakening. Moreover, other studies found combinations of different subtypes to be more strongly associated with depression than early morning awakening (Taylor et al., 2005; Espie et al., 2012). Even though research suggests that insomnia overall is more strongly related to anxiety than depression (Taylor et al., 2005), it appears to exist less research on the relationship between anxiety and insomnia subtypes. Regarding hypnotics, a previous study by Espie et al. (2012) found that patients reporting a mixed subtype of insomnia were more likely to be taking prescribed sleeping pills than other insomnia subtypes. Hohagen et al. (1994) reported similar results. Alcohol consumption is a common self-treatment strategy in patients with insomnia (Ohayon, 2002). Still, to our knowledge, few previous studies have investigated the distribution of alcohol consumption across subtypes of insomnia (Ohayon, 2002; Ebrahim et al., 2013; Riemann et al., 2017).

Considering previous research, the present study aimed to examine the distribution of demographic characteristics such as sex, age, level of education and marital status across subtypes of insomnia. We also aimed to investigate the prevalence of alcohol consumption, use of hypnotics, possible anxiety and possible depression within the different insomnia subtypes. We hypothesized that these parameters would be more prevalent when participants experienced a mixed insomnia subtype consisting of all three nocturnal insomnia symptoms (sleep onset insomnia, sleep maintenance insomnia and early morning awakening) compared to experiencing one or two of these separately.

## Materials and Methods

The present study was based on an extensive online interactive questionnaire made publicly available on the webpage of the Norwegian Competence Center for Sleep Disorders (www.sovno.no) in February 2012, following information about this in Norwegian newspapers and on a national television website. Staff at the competence center and others have later recommended people who suffer from sleep problems to complete the online questionnaire. After completing all questions, participants automatically receive feedback on the type of sleep disorder they may suffer from, and advice on how to deal with the problem. There are no specific inclusion or exclusion criteria. The data used in the present study was downloaded in January 2019, after 113 887 people had responded to parts of, or the entire questionnaire. A total of 3 309 participants reported that they previously had completed the survey, consequently their second responses were removed from the data. All data concerned participants who subjectively stated problems associated with sleeping.

Participants were asked about sex (male; female), age (response alternatives ranging from 15 to more than 101 years), level of education (primary school; secondary school; vocational school; university), marital status (married; single; cohabitant; divorced; separated; widow/widower) and circadian preference (definitely morning type; more morning type than evening type; neither morning type nor evening type; more evening type than morning type; definitely evening type). Participants were asked about alcohol consumption (yes; no). Those answering yes were further asked about the frequency of alcohol consumption with the following response alternatives «daily»; «3–5 days per week»; «1–2 days per week»; and «rarely». The questionnaire did not include a question about the amount of alcohol consumed. The participants were also asked if they currently use hypnotics (yes; no). We did not specify type of hypnotic, and whether they were prescribed or over-the-counter medications. The participants also reported the duration of their subjectively stated sleep problem and were provided the following response alternatives: “less than 6 months,” “6–12 months,” “1–2 years,” “2–3 years” etc., up to “more than 50 years.”

The Hospital Anxiety and Depression Scale (HADS) was used to assess symptoms of anxiety and depression during the last week. From a total of 14 statements, 7 of them aim to detect anxiety, while the remaining 7 statements aim to detect depression. Each statement has 4 response alternatives reflecting increasing severity (0–3). HADS ≥ 8 in the respective subscales was used to define possible anxiety and possible depression (Bjelland et al., 2002). In the present study, Cronbach’s alpha was 0.82 and 0.81 for HAD-anxiety and HAD-depression, respectively.

Bergen Insomnia Scale (BIS) was used to evaluate insomnia symptoms. BIS was initially developed according to the diagnostic criteria for insomnia used in the 4th edition of the Diagnostic and Statistical Manual of Mental Disorders (DSM-IV-TR) (Pallesen et al., 2008). The presence of a specific insomnia symptom the last month is scored along a scale indicating the number of days per week it is experienced (0–7 days). BIS consists of six items, of which the first three pertain to sleep onset, sleep maintenance, and early morning awakening, respectively. The participants are specifically asked if they used more than 30 min to fall asleep after the light was switched off, if they were awake for more than 30 min between periods of sleep, or if they awakened more than 30 min earlier than they wished without being able to fall asleep again. The last three items refer to not feeling adequately rested, experiencing daytime impairment and being dissatisfied with current sleep. The Cronbach’s alpha for the BIS was 0.70 in the present study.

Chronic insomnia disorder was defined according to the criteria from DSM-5 and the International Classification of Sleep Disorders (ICSD-3) as scoring three days per week or more on at least one of the first three items in addition to experiencing daytime impairment and/or dissatisfaction with sleep three days per week or more (American Psychiatric Association [APA], 2013; American Academy of Sleep Medicine, 2014). Participants who met the criteria for chronic insomnia disorder were further divided into subtypes. The main criterion to be diagnosed with “Sleep onset latency insomnia” (SOL-insomnia) was to have experienced sleep onset latency exceeding 30 min three days per week or more the last month. Analogously, the main criterion to be diagnosed with “Wake after sleep onset insomnia” (WASO-insomnia) was wakefulness exceeding 30 min between periods of sleep three days per week or more the last month, and the main criterion to be diagnosed with “Early morning awakening insomnia” (EMA-insomnia) was early morning awakening exceeding 30 min three days per week or more the last month. To be diagnosed with one of the three abovementioned subtypes the participant could not show symptoms related to the other two. Participants showing symptoms of more than one of the first three subtypes were divided into the following: SOL + WASO-insomnia was diagnosed if participants met the criteria of both SOL- and WASO-insomnia. SOL + EMA-insomnia was diagnosed in participants meeting the criteria of both SOL- and EMA-insomnia. EMA + WASO-insomnia was diagnosed in participants meeting the criteria of both EMA- and WASO-insomnia. SOL + WASO + EMA-insomnia was diagnosed if participants met all three main criteria. Thus, a total of seven specific subtypes of insomnia were defined.

### Ethics

No personally identifiable information was stored in the collected data file, thus the participants remained anonymous. The project leader (BB) requested ethical approval by the Ethics Board (The regional committee for Medical and Health Related Research Ethics in Western Norway), who exempted the survey from ethical review due to the anonymous nature of the study.

### Statistics

IBM SPSS Statistics, version 25.0 was used for the data analyses. Frequency analysis was conducted to provide a description of the study population. Distribution of the insomnia subtypes within the characteristics sex, age, education, marital status and circadian preference was explored using Pearson’s chi square tests. Furthermore, differences regarding use of alcohol, hypnotics, possible anxiety (HAD-A ≥ 8) and possible depression (HAD-D ≥ 8) according to each insomnia subtype were explored using the same statistics.

Separate logistic regression analyses were conducted with possible anxiety (HAD-A ≥ 8), possible depression (HAD-D ≥ 8), use of hypnotics, and alcohol consumption as dependent variables. The insomnia subtypes were used as predictors with SOL-insomnia as the reference (OR = 1.00). Reported alcohol consumption was dichotomized into high consumption (≥3 days per week) and low consumption (<3 days per week). The first analysis was unadjusted (crude), the second adjusted for age and sex, whereas the third analysis adjusted for education and marital status in addition to sex and age. Significance level was set to *P* < 0.05.

## Results

**FIGURE 1:**
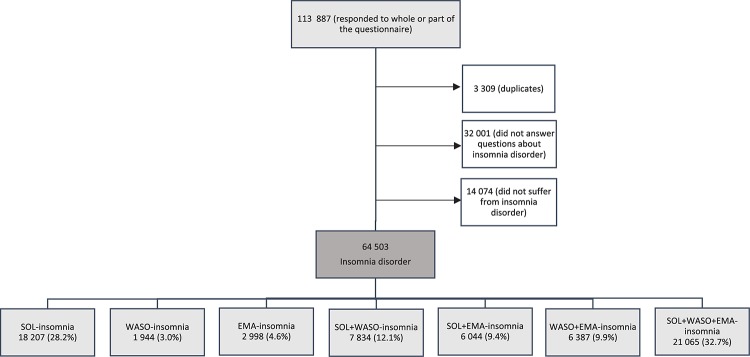
A total of 113 887 people responded to parts of, or the entire questionnaire. In all, 3 309 participants who reported that they previously had completed the survey, got their second responses removed from the data. A total of 78 577 participants answered questions about insomnia disorder, of which 64 503 (82.1%) fulfilled the insomnia criteria according to DSM-5. The prevalence of the specific insomnia subtypes among the participants who met the criteria for chronic insomnia disorder is shown: SOL, sleep onset; WASO, wake after sleep onset; EMA, early morning awakening.

A total of 78 577 participants answered questions about insomnia disorder, of which 64 503 (82.1%) fulfilled the insomnia criteria according to DSM-5, comprising the study population (Figure 1). Among these, 87.1% reported a duration of insomnia of six months or longer. Further characteristics of the study population are summarized in Table 1. Mean age was 36.9 years (SD = 14.6), with a preponderance of women (64.8%). A high level of education predominated (49.0%). The majority of the participants identified themselves as evening types, with 28.6% answering “more evening type than morning type” and 26.8% answering “definitely evening type.” The overall prevalence of possible anxiety and possible depression was high among the participants, with 63.3% and 39.2% having possible anxiety and possible depression, respectively.

**TABLE 1:** Characteristics of the participants with chronic insomnia disorder (*n* = 64 503).

	*n (%)*
***Sex: (missing 4)***	
Male	22717 (35.2)
Female	41782 (64.8)
***Age: (missing 3)***	
15–25 years	16417 (25.5)
26–35 years	15138 (23.5)
36–45 years	12940 (20.1)
46–55 years	11154 (17.3)
56–65 years	6328 (9.8)
Over 65 years	2523 (3.9)
***Level of education: (missing 5)***	
Primary school	7955 (12.3)
Secondary school or vocational school	24918 (38.6)
University or college	31625 (49.0)
***Marital status: (missing 5)***	
Single	24086 (37.3)
Married, cohabitant	35444 (54.9)
Divorced, separated, widow/widower	4968 (7.7)
***Circadian rhythm (missing 1 913)***	
Definitely a morning type	5838 (9.3)
More morning type than evening type	9773 (15.6)
Neither morning nor evening type	12355 (19.7)
More evening type than morning type	17880 (28.6)
Definitely an evening type	16744 (26.8)
***Alcohol consumption (missing 3 619)***	
No	20128 (33.1)
Rarely	18614 (30.6)
1–2 times per week	17286 (28.4)
3–5 times per week	4085 (6.7)
Daily	771 (1.3)
***Use of hypnotics (missing 3 703)***	
Yes	10529 (17.3)
No	50271 (82.7)
***Mental health (missing 1 439)***	
Possible anxiety (HAD-A ≥ 8)^a^	38949 (63.3)
Possible depression (HAD-D ≥ 8)^b^	24115 (39.2)
***Duration of sleep problems (missing 0)***	
<6 months	8307 (12.9)
≥6 months	56196 (87.1)

*^*a*^HAD-A = Hospital Anxiety and Depression Scale—Anxiety subscale. ^*b*^HAD-D = Hospital Anxiety and Depression Scale— Depression subscale.*

Table 2 presents the prevalence of the specific insomnia subtypes among the participants who met the criteria for chronic insomnia disorder. SOL + WASO + EMA-insomnia (32.7%) followed by SOL-insomnia occurring alone (28.2%) were the most common subtypes. All subtypes comprised more than 1900 participants. Table 2 also presents the distribution of the insomnia subtypes within selected demographic characteristics. As further detailed in the table, the prevalence of SOL + WASO + EMA-insomnia compared to the other subtypes was higher within females than within males. On the contrary, the distribution of SOL-insomnia compared to the other subtypes was lower within females than within males. Age was positively associated with SOL + WASO + EMA- insomnia, while inversely associated with the occurrence of SOL-insomnia. In the age group 15–25 years, 47.1% were diagnosed with SOL-insomnia, while 56.5% of participants 65 years or older were diagnosed with SOL + WASO + EMA-insomnia. Participants within all three levels of education showed a higher prevalence of SOL + WASO + EMA-insomnia than they did of any other insomnia subtype. Still, the highest proportion of SOL + WASO + EMA-insomnia was found among participants with the lowest level of education. Furthermore, single participants reported a higher prevalence of SOL-insomnia than of other insomnia subtypes, while participants who were married, cohabitant, divorced, separated or a widow/widower showed a higher prevalence of SOL + WASO + EMA-insomnia than of other insomnia subtypes. Again, the highest percentage of SOL + WASO + EMA-insomnia was found among the participants who were divorced, separated or a widow/widower. Being an evening type was clearly associated with SOL-insomnia. Being a morning type was more commonly reported by participants with SOL + WASO + EMA-insomnia.

**TABLE 2:** The percentage distribution of the specific insomnia subtypes within selected demographic parameters (*n* = 64 503).

	*SOL-*	*WASO-*	*EMA-*	*SOL* +	*SOL* +	*WASO* +	*SOL* + *WASO* +	*Chi-square*	*p-value^b^*
	*insomnia*	*insomnia*	*insomnia*	*WASO-insomnia*	*EMA-insomnia*	*EMA-insomnia*	*EMA-insomnia*	*(df)^a^*	
% (n)	*28.2 (18207)*	*3.0 (1944)*	*4.6 (2998)*	*12.1 (7834)*	*9.4 (6044)*	*9.9 (6387)*	*32.7 (21 065)*		
***Sex***									
Male	30.4%	2.8%	5.6%	11.1%	8.7%	10.5%	30.9%	232.2 (6)	<0.0005
Female	27.1%	3.2%	4.1%	12.7%	9.7%	9.6%	33.6%		
***Age***									
15–25 years	47.1%	1.4%	2.4%	14.1%	12.1%	1.8%	21.1%	11102.4 (30)	<0.0005
26–35 years	37.2%	2.9%	4.2%	13.6%	10.5%	5.2%	26.4%		
36–45 years	21.6%	3.9%	6.2%	11.8%	8.9%	12.4%	35.1%		
46–55 years	11.7%	4.2%	6.4%	9.8%	7.2%	18.9%	41.8%		
56–65 years	9.2%	3.6%	5.5%	10.0%	6.2%	18.7%	46.9%		
>65 years	6.6%	3.2%	3.9%	8.5%	4.8%	16.4%	56.5%		
***Level of education***									
Primary school	29.1%	1.9%	3.5%	13.1%	10.7%	5.5%	36.2%	758.0 (12)	<0.0005
Secondary school or vocational school	28.3%	2.5%	4.2%	12.0%	10.0%	8.2%	34.7%		
University or college	28.0%	3.7%	5.3%	12.1%	8.5%	12.4%	30.1%		
***Marital status***									
Married, cohabitant	22.3%	3.7%	5.7%	11.5%	8.3%	13.3%	35.2%	4008.4 (12)	<0.0005
Single	39.4%	2.0%	3.1%	13.5%	11.0%	4.1%	26.9%		
Divorced, separated, widow/widower	16.9%	3.3%	4.3%	10.4%	8.8%	13.5%	42.8%		
***Circadian rhythm***									
Definitely a morning type	5.8%	3.9%	10.1%	4.7%	7.8%	26.9%	40.9%	10314.7 (24)	<0.0005
More morning type than evening type	2.7%	4.3%	7.6%	8.8%	9.0%	18.1%	39.5%		
Neither morning nor evening type	20.5%	3.4%	4.9%	11.5%	9.5%	10.5%	39.7%		
More evening type than morning type	32.3%	2.8%	3.6%	13.7%	10.6%	6.3%	30.6%		
Definitely an evening type	46.8%	2.0%	2.0%	15.6%	8.6%	2.7%	22.3%		

*SOL-insomnia = Sleep onset latency insomnia, WASO-insomnia = Wake after sleep onset insomnia, EMA-insomnia = Early morning awakening insomnia. Percentages adding up to 100% in each row. ^*a*^Degrees of freedom, ^*b*^Pearson Chi-square.*

As presented in Table 3, WASO + EMA-insomnia was associated with more frequent alcohol consumption (10.2% reporting consumption of alcohol 3–5 times per week and 2.6% reporting a daily consumption), while SOL-insomnia was associated with the least frequent consumption (4.9% reporting consumption of alcohol 3–5 times per week and 0.6% reporting a daily consumption). SOL + WASO + EMA-insomnia was associated with the highest use of hypnotics (26.8%) in comparison to the other subtypes. All insomnia subtypes were clearly associated with possible anxiety (50.8–68.1%) with WASO-insomnia and SOL + EMA-insomnia representing the lowest and highest association, respectively. Out of all insomnia subtypes, SOL + WASO + EMA-insomnia was associated with the highest prevalence of possible depression (46.0%), whereas WASO-insomnia was associated with the lowest (32.5%).

**TABLE 3:** The percentage distribution of alcohol consumption, hypnotic use, and mental health within each specific insomnia subtype (*n* = 64 503).

	*SOL-*	*WASO-*	*EMA-*	*SOL* + *WASO-*	*SOL* + *EMA-*	*WASO* + *EMA-*	*SOL* + *WASO* +	*Chi-square*	*p-value^d^*
	*insomnia*	*insomnia*	*insomnia*	insomnia	-insomnia	-insomnia	*EMA-insomnia*	*(df)^c^*	
***Alcohol consumption***									
No	29.7%	32.7%	33.9%	33.1%	34.9%	31.4%	35.9%	901.7 (24)	<0.0005
Rarely	35.3%	30.4%	30.5%	31.3%	33.4%	25.1%	27.1%		
1–2 times per week	29.6%	26.7%	26.9%	28.5%	26.1%	30.7%	27.6%		
3–5 times per week	4.9%	8.6%	7.2%	6.4%	4.8%	10.2%	7.6%		
Daily	0.6%	1.7%	1.4%	0.8%	0.8%	2.6%	1.7%		
***Use of hypnotics***									
Yes	11.4%	7.6%	8.6%	16.2%	16.6%	12.5%	26.8%	2046.0 (6)	<0.0005
No	88.6%	92.4%	91.4%	83.8%	83.4%	87.5%	73.2%		
***Mental health***									
Possible anxiety (HAD-A ≥ 8)^a^	61.6%	50.8%	55.8%	65.3%	68.1%	54.5%	67.7%	656.8 (6)	<0.0005
Possible depression (HAD-D ≥ 8)^b^	34.0%	32.5%	34.1%	40.4%	41.1%	33.3%	46.0%	756.8 (6)	<0.0005

*SOL-insomnia = Sleep onset latency insomnia, WASO-insomnia = Wake after sleep onset insomnia, EMA-insomnia = Early morning awakening insomnia. Percentages adding up to 100% in each column. ^*a*^HAD-A = Hospital Anxiety and Depression Scale—Anxiety subscale, ^*b*^HAD-D = Hospital Anxiety and Depression Scale—Depression subscale, ^*c*^Degrees of freedom, ^*d*^Pearson Chi-square.*

The results from the logistic regressions with possible anxiety, possible depression, alcohol consumption and use of hypnotics as dependent variables are presented in Table 4.

**TABLE 4:** Logistic regression analyses with possible anxiety, possible depression, hypnotic use, and alcohol consumption as dependent variables, and the specific insomnia subtypes as predictors in a Norwegian web-based study (*n* = 64 503).

	Possible anxiety (HAD-A^a^ ≥ 8)			Possible depression (HAD-D^b^ ≥ 8)		
	Crude OR	Adjusted OR^c^	Adjusted OR^d^	Crude OR	Adjusted OR^c^	Adjusted OR^d^
	**(95% CI)**	**(95% CI)**	**(95% CI)**	**(95% CI)**	**(95% CI)**	**(95% CI)**
SOL-insomnia	1.00	1.00	1.00	1.00	1.00	1.00
WASO-insomnia	**0.64 (0.58–0.71)**	**0.84 (0.76–0.93)**	**0.85 (0.77–0.93)**	0.94 (0.85–1.04)	1.08 (0.97–1.20)	1.11 (1.00–1.23)
EMA-insomnia	**0.79 (0.73–0.85)**	1.06 (0.97–1.15)	1.06 (0.98–1.16)	1.00 (0.92–1.09)	**1.13 (1.04–1.23)**	**1.15 (1.06–1.26)**
SOL + WASO-insomnia	**1.17 (1.11–1.24)**	**1.33 (1.26–1.41)**	**1.32 (1.25–1.40)**	**1.32 (1.24–1.39)**	**1.41 (1.33–1.49)**	**1.40 (1.32–1.48)**
SOL + EMA-insomnia	**1.33 (1.25–1.42)**	**1.47 (1.38–1.57)**	**1.45 (1.36–1.55)**	**1.35 (1.27–1.44)**	**1.43 (1.35–1.52)**	**1.40 (1.31–1.49)**
WASO + EMA-insomnia	**0.75 (0.70–0.79)**	**1.15 (1.08–1.23)**	**1.17 (1.10–1.25)**	0.97 (0.91–1.03)	**1.18 (1.11–1.26)**	**1.23 (1.15–1.31)**
SOL + WASO + EMA-insomnia	**1.31 (1.25–1.36)**	**1.79 (1.71–1.88)**	**1.76 (1.68–1.84)**	**1.66 (1.59–1.73)**	**1.93 (1.84–2.02)**	**1.87 (1.79–1.96)**

	**Use of hypnotics (yes)**			**Alcohol consumption (>3 days per week)**		
	**Crude OR**	**Adjusted OR^c^**	**Adjusted OR^d^**	**Crude OR**	**Adjusted OR^c^**	**Adjusted OR^d^**

	**(95% CI)**	**(95% CI)**	**(95% CI)**	**(95% CI)**	**(95% CI)**	**(95% CI)**
SOL-insomnia	1.00	1.00	1.00	1.00	1.00	1.00
WASO-insomnia	**0.64 (0.54–0.77)**	**0.40 (0.33–0.48)**	**0.40 (0.34–0.48)**	**1.97 (1.67–2.32)**	**1.23 (1.03–1.45)**	1.14 (0.97–1.36)
EMA-insomnia	**0.73 (0.64–0.84)**	**0.47 (0.41–0.55)**	**0.48 (0.42–0.55)**	**1.63 (1.41–1.88)**	0.98 (0.84–1.14)	0.94 (0.81–1.09)
SOL + WASO-insomnia	**1.51 (1.40–1.63)**	**1.20 (1.11–1.30)**	**1.20 (1.11–1.30)**	**1.34 (1.20–1.49)**	1.06 (0.95–1.19)	1.06 (0.95–1.19)
SOL + EMA-insomnia	**1.55 (1.42–1.68)**	**1.31 (1.20–1.42)**	**1.29 (1.19–1.41)**	1.02 (0.90–1.17)	0.85 (0.75–0.97)	0.88 (0.77–1.01)
WASO + EMA-insomnia	**1.11 (1.02–1.22)**	**0.59 (0.54–0.65)**	**0.60 (0.55–0.66)**	**2.53 (2.29–2.79)**	**1.26 (1.13–1.40)**	**1.18 (1.06–1.32)**
SOL + WASO + EMA-insomnia	**2.86 (2.70–3.02)**	**1.81 (1.70–1.92)**	**1.80 (1.69–1.91)**	**1.78 (1.64–1.93)**	1.05 (0.96–1.14)	**1.10 (1.00–1.20)**

*SOL-insomnia = Sleep onset latency insomnia, WASO-insomnia = Wake after sleep onset insomnia, EMA-insomnia = Early morning awakening insomnia, 95% CI = 95% confidence interval. ^*a*^HAD-A = Hospital Anxiety and Depression Scale—Anxiety subscale, ^*b*^HAD-D = Hospital Anxiety and Depression Scale—Depression subscale, ^*c*^Adjusted for sex and age, ^*d*^Adjusted for sex, age, level of education and marital status.*

Both crude and adjusted logistic regressions with possible anxiety as the dependent variable showed a negative association with WASO-insomnia compared to SOL-insomnia. Both EMA-insomnia and WASO + EMA-insomnia were negatively associated with possible anxiety compared to SOL-insomnia in the crude analyses. While EMA-insomnia was no longer significantly associated with possible anxiety compared to SOL-insomnia in the adjusted analyses, WASO + EMA-insomnia became positively associated. SOL + WASO-insomnia, SOL + EMA-insomnia and SOL + WASO + EMA-insomnia were all stronger predictors of possible anxiety than SOL-insomnia, with SOL + WASO + EMA-insomnia being the strongest predictor in the adjusted analyses. Furthermore, in the crude analysis, SOL + WASO-insomnia, SOL + EMA-insomnia and SOL + WASO + EMA-insomnia were all stronger predictors of possible depression than SOL-insomnia, with SOL + WASO + EMA-insomnia being the strongest. Compared to SOL-insomnia, all insomnia subtypes except WASO-insomnia were positively associated with possible depression in the adjusted analyses, with SOL + WASO + EMA-insomnia being the strongest predictor (fully adjusted OR = 1.87).

Compared to SOL-insomnia, WASO-insomnia and EMA-insomnia were inversely associated with use of hypnotics in both the crude and adjusted analyses. WASO + EMA-insomnia was positively associated with use of hypnotics in the crude analysis, while negatively associated in both adjusted analyses. SOL + WASO-insomnia, SOL + EMA-insomnia and SOL + WASO + EMA-insomnia were all stronger predictors of use of hypnotics relative to SOL-insomnia, with SOL + WASO + EMA-insomnia remaining the strongest predictor in both crude (OR = 2.86) and the two adjusted (fully adjusted OR = 1.80) analyses. Compared to SOL-insomnia, all insomnia subtypes, except SOL + EMA-insomnia, were positively associated with alcohol consumption three or more days per week in the crude analysis. However, in the fully adjusted model, only WASO + EMA-insomnia and SOL + WASO + EMA-insomnia remained positively associated with high alcohol consumption.

## Discussion

In total more than 60% of the participants with chronic insomnia disorder had either SOL-insomnia or SOL + WASO + EMA-insomnia. These two insomnia subtypes differed considerably regarding age. SOL-insomnia was clearly the most common insomnia subtype among younger participants. On the contrary, more than half of the oldest participants reported SOL + WASO + EMA-insomnia. The distribution of insomnia subtypes within the other demographic characteristics showed that participants with female sex, low level of education and who were divorced, separated or a widow/widower had a higher prevalence of SOL + WASO + EMA-insomnia compared to participants with male sex, high level of education and who were married, cohabitant or single, respectively. Almost all epidemiological studies have reported an increased prevalence of insomnia symptoms with female sex, age and when being separated, divorced or a widow/widower and having a low level of education (Ohayon, 2002). Our findings further contribute to the research area by adding knowledge about the distribution of these characteristics within different insomnia subtypes, particularly their strong association with SOL + WASO + EMA-insomnia.

Moreover, our findings indicated that possible anxiety and possible depression more frequently occurred in participants with SOL + WASO + EMA-insomnia compared with SOL-insomnia. While this is somewhat in contrast with previous studies suggesting depression to be more strongly associated with early morning awakening (Rodin et al., 1988; Lustberg and Reynolds, 2000) or difficulty initiating sleep (Yokoyama et al., 2010; Ikeda et al., 2017), it is important to emphasize that previous studies did not include mixed insomnia subtypes. It can therefore not be ruled out that these studies would have provided similar results as the current study if they had also included combinations of the insomnia subtypes. Based on our results we argue that SOL + WASO + EMA-insomnia has a greater impact on daily life compared to the other subtypes, considering the especially close relationship between possible anxiety and possible depression and SOL + WASO + EMA-insomnia. This assumption seems conceivable as this insomnia subtype should be regarded as the most serious as it implies sleeplessness in all temporal phases of the main sleep episode. Espie et al. (2012) support this finding by suggesting that participants with a combination of insomnia symptoms have greater impairment in several important areas of daily functioning, particularly energy and mood. Taylor et al. (2005) suggested that people with combined insomnia had the highest depression score, while they found no differences on anxiety scores between insomnia subtypes, thereby partly supporting the findings of the current study. It is worth mentioning that possible anxiety was least frequent in participants experiencing WASO-insomnia. We assume that this finding could be explained by the nature of anxiety as worrying while still awake in the evening and after waking up in the morning, where both would disturb the ability to sleep (Clancy et al., 2020).

Our assumption about alcohol was inconsistent with our findings. WASO + EMA-insomnia was associated with the most frequent alcohol consumption. This may be explained by alcohol’s effect on sleep patterns, where alcohol at bedtime may facilitate sleep onset, but typically causes sleep disruption in the second half of the sleep period (Ohayon, 2002; Ebrahim et al., 2013). However, alcohol loses its effect on sleep onset, while sleep disruption remains when consumed frequently (Ohayon, 2002). This may explain why SOL + WASO + EMA-insomnia also was associated with a high alcohol consumption in the fully adjusted logistic regression. Drinking quantity and frequency are previously reported to be independent predictors of insomnia (Kim et al., 2017). It is also found that patients with alcohol abuse problems have significantly higher insomnia scores than those without (Atalay, 2011). Still, alcohol is often used as a self-treatment strategy in the general population (Ohayon, 2002). Indeed, some longitudinal studies have attested to the assumption that insomnia may be a risk factor for future problematic alcohol consumption (Hasler et al., 2014). Conversely, longitudinal studies have also shown that quantity of drinking may predict future insomnia severity (Zhabenko et al., 2013). Based on the current literature it seems reasonable to assume a bi-directional relationship between insomnia and alcohol consumption. Regarding insomnia subtypes, Edinger et al. (1996) found that patients suffering from insomnia associated with alcohol use complained of both sleep onset insomnia and sleep maintenance insomnia (including wake after sleep onset and early morning awakening), and that these patients had the longest histories of insomnia complaints compared to other patients. However, that finding is based on a cluster consisting of 5 patients only (Edinger et al., 1996).

As for use of hypnotics, our assumption was consistent with our findings. SOL + WASO + EMA-insomnia was clearly associated with the highest use of hypnotics, which may be another representation of the greater impact on daily life experienced by participants with this insomnia subtype or reflecting the fact that this subtype is the most burdensome in terms of the nocturnal symptoms. The subtypes that did not include sleep onset problems were less associated with use of hypnotics than SOL-insomnia in the adjusted logistic analyses. Our findings therefore suggest that prolonged sleep onset may be a potent antecedent for use of hypnotics. The efficacy of pharmacological therapy for chronic sleep onset insomnia was evaluated in a randomized, placebo-controlled clinical trial and the finding suggests that patients with sleep onset insomnia can derive significantly greater benefit from cognitive behavioral therapy (CBT-I) than pharmacotherapy (Jacobs et al., 2004). This is consistent with the most recent guidelines considering CBT-I as the first-line treatment for chronic insomnia disorder (Riemann et al., 2017). Despite this, CBT-I is not well known by health care practitioners and remains underused in clinical practice. Insomnia is thereby undertreated, and hypnotics are prescribed too often for long-term use, possibly leading to tolerance and a rebound effect at discontinuation (Ohayon, 2002).

The present study has several limitations that should be noted. The prevalence of SOL-insomnia gradually decreased with age in the present study. The reported circadian preference seemed to be a contributing factor to these findings. This emphasizes a possible limitation of the present study, where other sleep disorders such as a circadian rhythm sleep-wake disorder may be difficult to differentiate from an insomnia disorder. In this regard it should be noted that being an evening type was clearly associated with SOL-insomnia. Younger participants not being able to fall asleep in the evening could in some cases therefore be suffering from a circadian rhythm sleep-wake disorder (delayed sleep-wake phase disorder), rather than insomnia disorder (Hysing et al., 2013; Alvaro et al., 2014; Gupta et al., 2014; Benjamins et al., 2017; Riemann et al., 2017; Blake et al., 2018). A potential new study should address this issue by including more questions related to circadian rhythm sleep-wake disorders. Questions regarding drug abuse, other pharmacological treatments and symptoms of disorders beyond anxiety and depression were not included in the current study. Thus, future studies should investigate a wider array of potential covariates in terms of their associations with insomnia subtypes. While the high prevalence of SOL-insomnia found in younger participants could mirror a high rate of true insomnia, it could thus also reflect the need for adjustment of some of the criteria for SOL-insomnia. Nevertheless, the cut-off for SOL-insomnia of 30 min or more is based on epidemiological studies of adults (Hysing et al., 2013). In addition, by adjusting for relevant factors such as sex and age we still found significant associations between the different insomnia subtypes and possible anxiety, possible depression and use of alcohol and hypnotics. This information is useful for clinicians in terms of understanding insomnia comorbidity and may thus also have treatment implications.

Another limitation to consider concerns the duration criteria used in BIS (one month), which is based on the criteria found in the DSM-IV. In DSM-5, the diagnosis of chronic insomnia disorder requires symptoms to last for at least 3 months. However, among the participants in our study 87.1% reported a duration of insomnia symptoms for six months or longer. We therefore argue that the used duration criteria most likely had minor impacts on the results. In addition, both the BIS and the HADS are considered standardized and validated instruments. BIS is regarded as a well validated questionnaire with good psychometric properties (Pallesen et al., 2008; Riemann et al., 2017), and HADS is found to have sensitivities and specificities of approximately 0.80 for both subscales using the cut-off used in the present study (Bjelland et al., 2002). It is also worth mentioning that HADS does not include sleep disturbance items, thereby precluding tautology when assessing the relationship with sleep variables (Neckelmann et al., 2007). Still, it should be noted that neither the BIS nor HADS provide a clinical diagnosis of insomnia, anxiety or depression, respectively.

Another limitation to consider is the concern first raised by Hohagen et al. (1994) who reported variability in insomnia subtypes over time. In their study, no more than about half of the patients remained classified with the same insomnia subtype at baseline and at the 4-month follow-up. Regardless of temporal stability, treatment based on insomnia subtype can be initiated at the time of diagnosis and then be changed if the insomnia subtype changes. Change in insomnia subtype over time may as well reflect treatment-response. Future studies should nevertheless include at least one follow-up in order to assess the temporal stability of insomnia subtypes.

The common methodological disadvantages of a self-report questionnaire-based study such as recall bias, common method bias and social desirability bias should further be considered as limitations (Podsakoff et al., 2003). In future studies these limitations may be overcome by using clinical interviews instead of questionnaires. A final limitation to consider is the possibility of selection bias. However, the possible selection bias is considered less significant when adjusting for relevant factors. In addition, we argue that several of the aforementioned limitations are mitigated by the large sample size achieved in the present study. As mentioned, all insomnia subtypes had a sample size of more than 1900 participants. To the best of our knowledge, the present study is the largest to date, which we consider its most important asset.

The present study could help clinical practitioners to tailor treatment approach based on characteristics and comorbidity of insomnia subtypes. To be able to do this, we argue that there is a need for a simple and clinically applicable classification of subtypes of insomnia. To be clinically applicable, we divided into insomnia subtypes based on their temporal association with the main sleep period. Our results suggested that insomnia is a heterogenic disorder and that there are differences between the insomnia subtypes’ effect on daily life. Participants with SOL + WASO + EMA-insomnia seemed to be suffering from a higher burden than participants with the other insomnia subtypes. They were more likely to have possible anxiety and depression, high alcohol consumption and hypnotic use compared to those suffering from other insomnia subtypes. Further research is required to determine whether tailored treatment in line with the insomnia subtypes will have clinical consequences in terms of treatment response.

## Data Availability Statement

The datasets generated for this study are available on request to the corresponding author.

## Ethics Statement

Ethical review and approval was not required for the study on human participants in accordance with the local legislation and institutional requirements. Written informed consent from the participants’ legal guardian/next of kin was not required to participate in this study in accordance with the national legislation and the institutional requirements.

## Author Contributions

IB and VJ designed the study, analyzed the data, and co-authored the manuscript. BB and SP collected the data, designed the study, interpreted the data, and reviewed the paper. All authors contributed to manuscript revision, read and approved the submitted version.

## Conflict of Interest

The authors declare that the research was conducted in the absence of any commercial or financial relationships that could be construed as a potential conflict of interest.
